# Supplementary material for: Oncolytic adeno-immunotherapy modulates the immune system enabling CAR T-cells to cure pancreatic tumors
Source: Commun Biol. 2021 Mar 19;4:368. doi: 10.1038/s42003-021-01914-8 (PMC7979740; doi:10.1038/s42003-021-01914-8)
Supplement: Supplementary file 2 — Supplementary Information [file 42003_2021_1914_MOESM2_ESM.pdf]

SUPPLEMENTARY INFORMATION

Oncolytic adeno-immunotherapy modulates the immune system enabling CAR T-cells to cure pancreatic tumors

Rosewell Shaw et al.

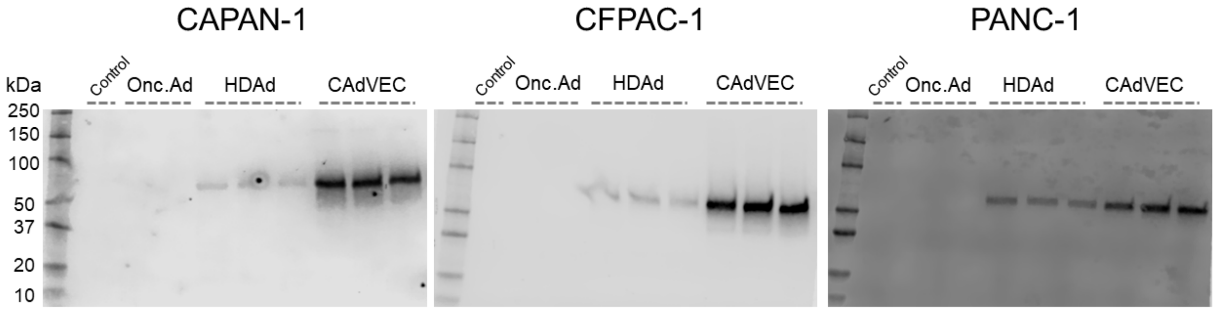

Supplementary Figure 1. *Co-infection of OAd amplifies PD-L1 mini-antibody secretion*. PANC-1, CAPAN-1 and CFPAC-1 were infected with total 10 vp/cell of HDAd*Trio*, OAd or CAd*Trio* (OAd:HDAd=1:1) (n=4 biologically independent samples). We sampled media 48 hours post-infection and quantified levels of PD-L1 mini-antibody by western blotting for PD-L1 mini-antibody.

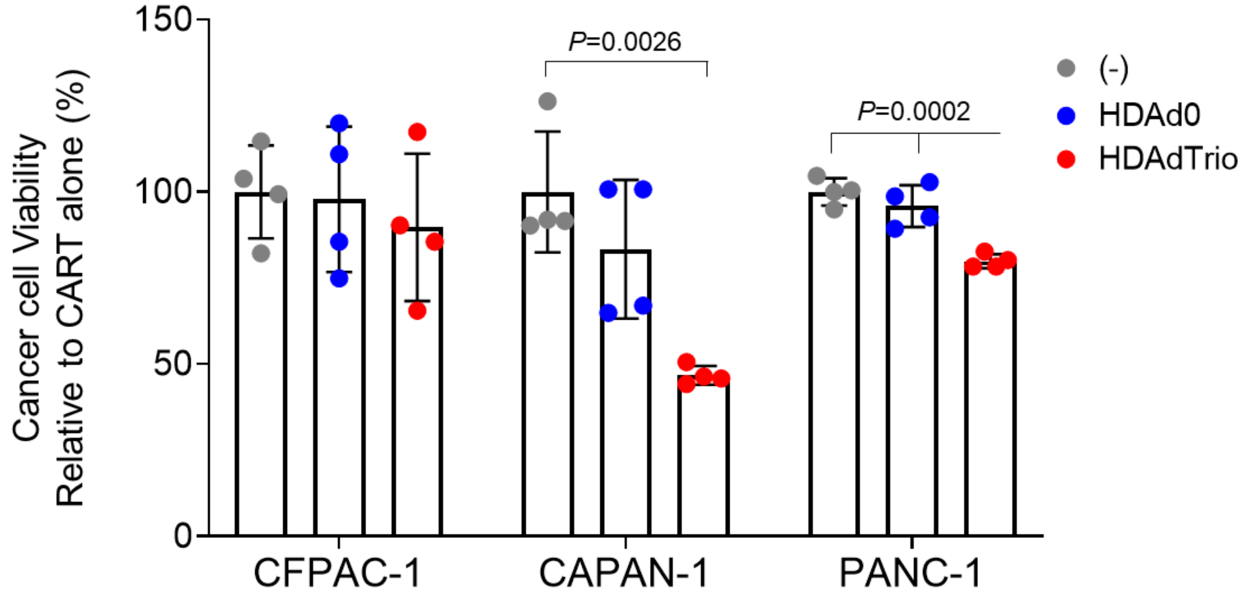

Supplementary Figure 2. ***Immunostimulatory molecules derived from CAdTrio enhance HER2.CART anti-tumor efficacy***. PANC-1, CAPAN-1 and CFPAC-1 expressing *ffLuc* were infected with 100 vp/cell of HDAd0 (no transgene) or HDAd*Trio*. Cells were cultured with HER2.CARTs (E:T=1:40) at 24 hours post-infection. Cells were harvested 72 hours post-coculture, and viable cancer cells were analyzed by luciferase assay (n=4 independent biological samples). Data were normalized based on viability of HER2.CART alone. Data are presented as means ± SD. P-values were determined by ordinary one-way ANOVA with Tukey multiple comparisons, p=0.0026 (F2,9)=12.35, p=0.0002 (F2,9)=29.36). Statistical significance set at $p < 0.05$, ns > 0.05.

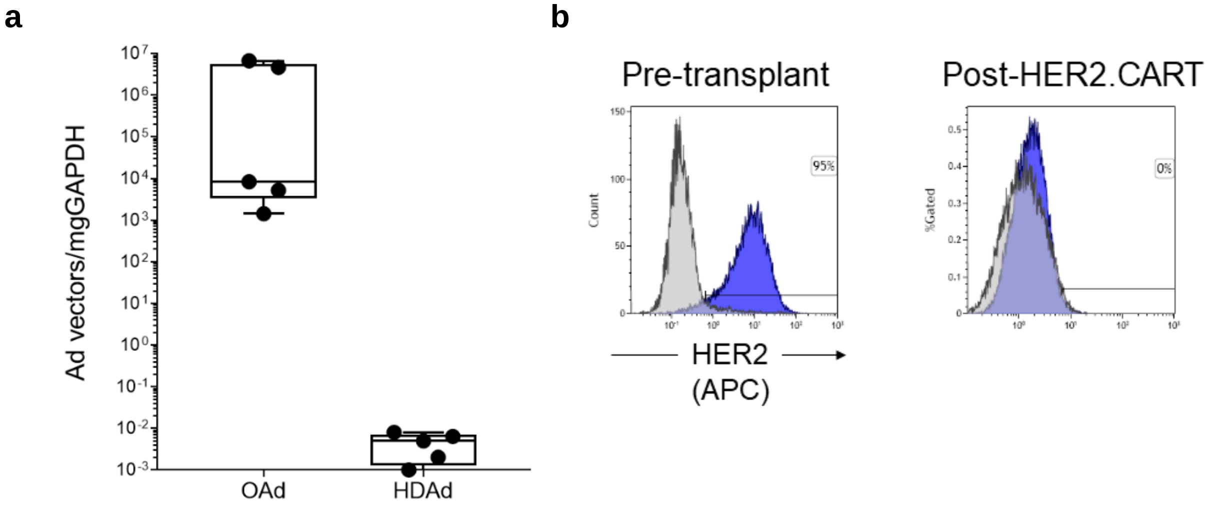

Supplementary Figure 3. ***Ad vectors persist at CAPAN-1 tumor site but do not control growth, and CAPAN-1 tumor causes antigen escape after HER2.CART treatment.*** **(a)** CAPAN-1 tumors treated with CAd*Trio* alone were harvested when tumor volume reached >1,500 mm^3. DNA was extracted from each tumor sample (n=5 animals), and the copy number of each Ad vector determined by quantitative PCR. Data was normalized with murine genomic GAPDH. Box plot elements: central line, median; box limit, upper and lower quartile; whisker, 1.5x inter-quartile range; points, outliers. **(b)** CAPAN-1 tumor treated with HER2.CART alone was harvested at the end of experiment (80 days post-infusion of HER2.CART), and HER2 expression of residual tumor was analyzed by flow cytometry.

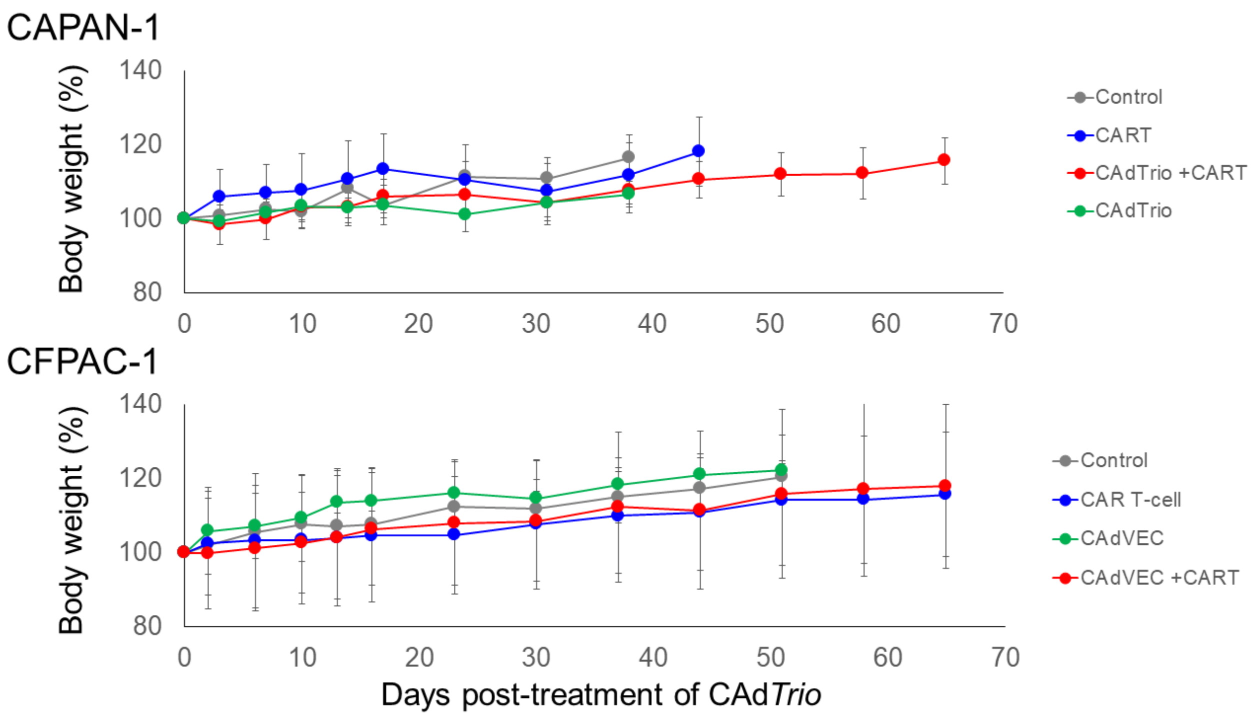

Supplementary Figure 4. ***Combinatorial immunotherapy did not cause weight loss in xenograft mice.*** CAPAN-1 or CFPAC-1 cells were transplanted into the right flanks of NSG mice. A total of $1x10^7$ vp of CAd*Trio* (Onc:HD=1:1) were injected intra-tumorally. A total of $1x10^6$ HER2.CAR T-cells were systemically administered 3 days post-injection of CAd*Trio* (n=5 animals). Body weights were measured at different time points. Data are presented as means ± SD.

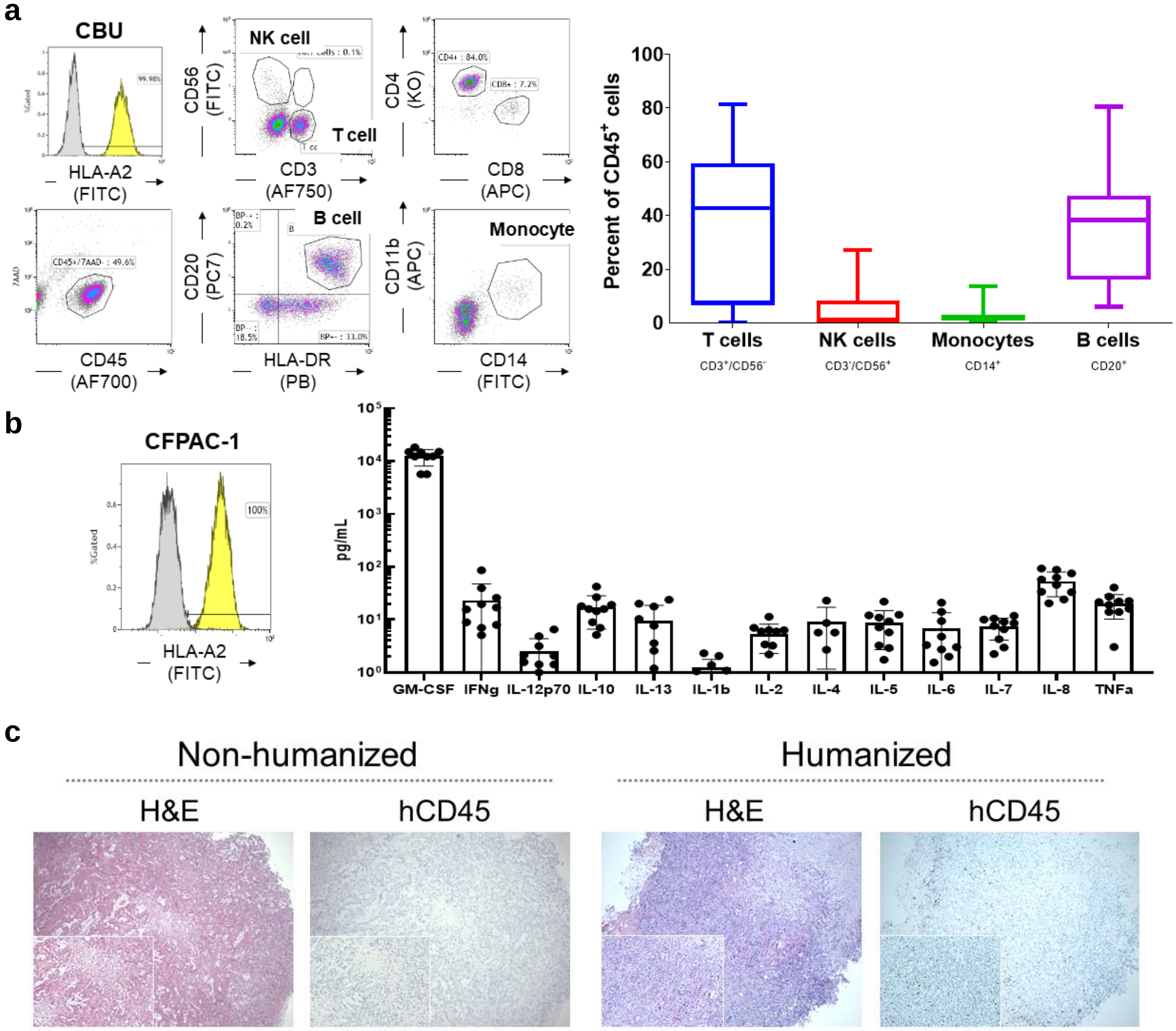

Supplementary Figure 5. ***Humanized mice reconstituted both innate and adaptive immune cells without inflammatory responses to transplanted CFPAC-1 cells*****.** **(a)** Human immune subsets in PBMCs of humanized mice (n=30 animals) were characterized by flow cytometry 10 weeks post-transplant of HLA-A2^{+} CBU derived CD34+ cells. Box plot elements: central line, median; box limit, upper and lower quartile; whisker, 1.5x inter-quartile range; points, outliers. **(b)** HLA-A2 expression on CFPAC-1 was analyzed by flow cytometry. Plasma samples were collected from humanized mice transplanted with CFPAC-1 at 17 days post-transplant and circulating human Th1 and Th2 cytokines were measured. **(c)** CFPAC-1 cells were transplanted into the right flanks of non-humanized and humanized mice. Tumors were fixed at 21 days post-transplant, and sections were stained with anti-human CD45 IgG.

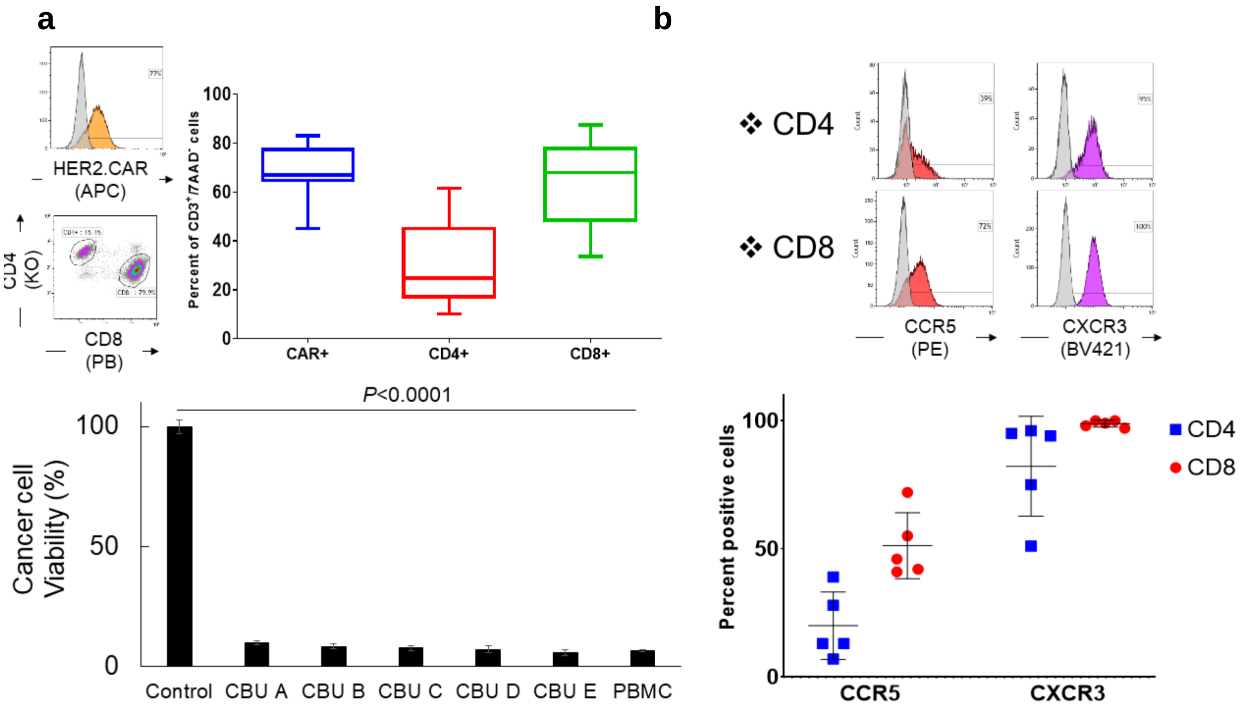

Supplementary Figure 6. ***Characterization of CBU derived HER2.CARTs*****. (a)** HER2.CAR expression and T cell subsets of CBU derived HER2.CARTs (n=20 independent biological samples) were analyzed by flow cytometry. Box plot elements: central line, median; box limit, upper and lower quartile; whisker, 1.5x inter-quartile range; points, outliers. CFPAC-1 cells expressing *ffLuc* were cultured with 5 different CBU-derived HER2.CARTs or healthy donor PBMC-derived HER2.CARTs (E:T=1:10). Cells were harvested 72 hours post-coculture, and viable cancer cells were analyzed by luciferase assay (n=4 independent biological samples). Data are presented as means ± SD. P-values were determined by ordinary one-way ANOVA with Tukey multiple comparisons, $p<0.0001$ ($F_{1.084,3.252}$)=2242). Statistical significance set at $p < 0.05$, ns > 0.05. **(b)** Chemokine receptors CCR5 and CXCR3 expressions were analyzed by flow cytometry on CBU derived HER2.CARTs (n=5).

❖56 days post-injection of CAdTrio

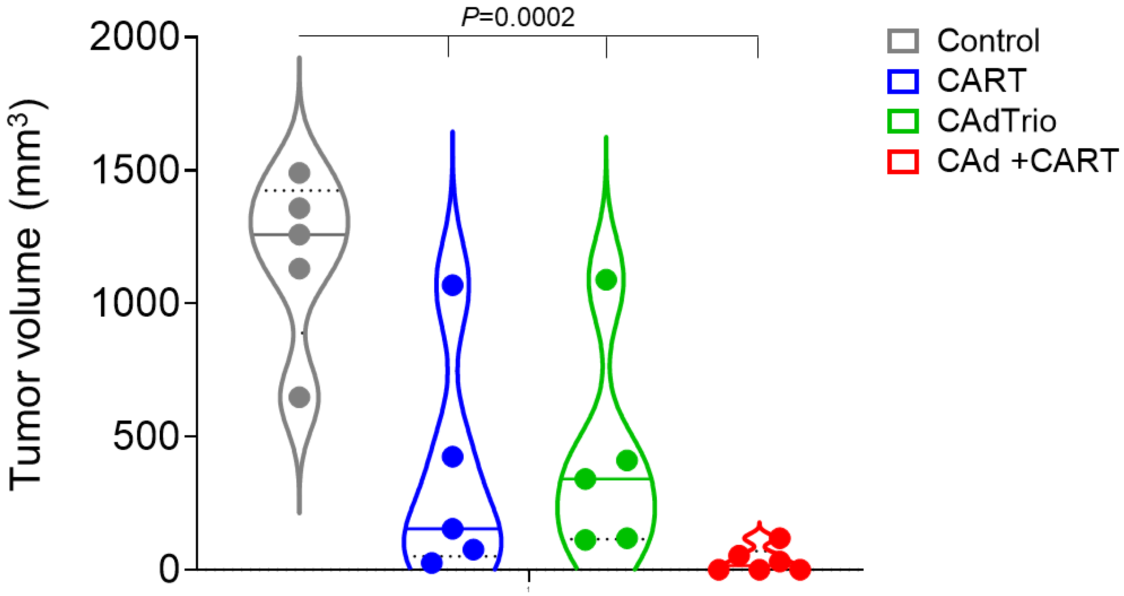

Supplementary Figure 7. ***Combination immunotherapy significantly controls CFPAC-1 tumor growth in humanized mice.*** CFPAC-1 cells were transplanted into the right flank of humanized mice (Control, CART alone, CAdTrio alone: n=5 animals, CAdTrio+CART: n=6 animals). A total of $1x10^7$ vp of *CAdTrio* (Onc:HD=1:1) were injected into the tumor. A total of $1x10^6$ HER2.CARTs expressing *ffLuc* were systemically administered 3 days post-injection of CAd*Trio*. Tumor volumes shown here from day 56 post injection of CAd, individual data points are represented in violin plot with means ± SD, p=0.0002. P-values were determined using ordinary one-way ANOVA with Tukey multiple comparisons, ($F(3,17)=11.66$). Statistical significance set at $p < 0.05$, ns > 0.05.

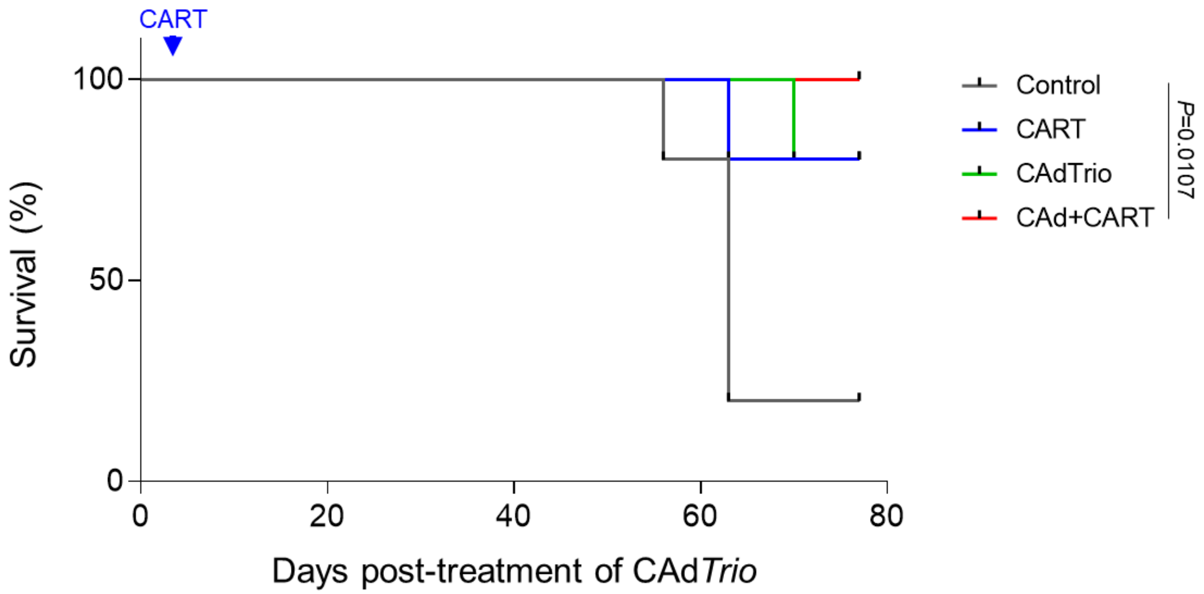

Supplementary Figure 8. *Immunotherapy components control CFPAC-1 tumor growth in humanized mouse model*. CFPAC-1 cells were transplanted into the right flank of humanized mice (Control, CART alone, CAdTrio alone: n=5 animals, CAdTrio+CART: n=6 animals). A total of $1x10^7$ vp of *CAdTrio* (OAd:HD=1:1) were injected into the tumor. A total of $1x10^6$ HER2.CARTs expressing *ffLuc* were systemically administered 3 days post-injection of CAd*Trio*. Kaplan-Meier survival curve based on the timepoint at which tumor volumes necessitated euthanasia (> 1,500 mm3) after CAd*Trio* administration in mice, p=0.0107. P-values were determined using the Log-rank Mantel-Cox test (dF=3). Statistical significance set at $p < 0.05$, ns > 0.05.

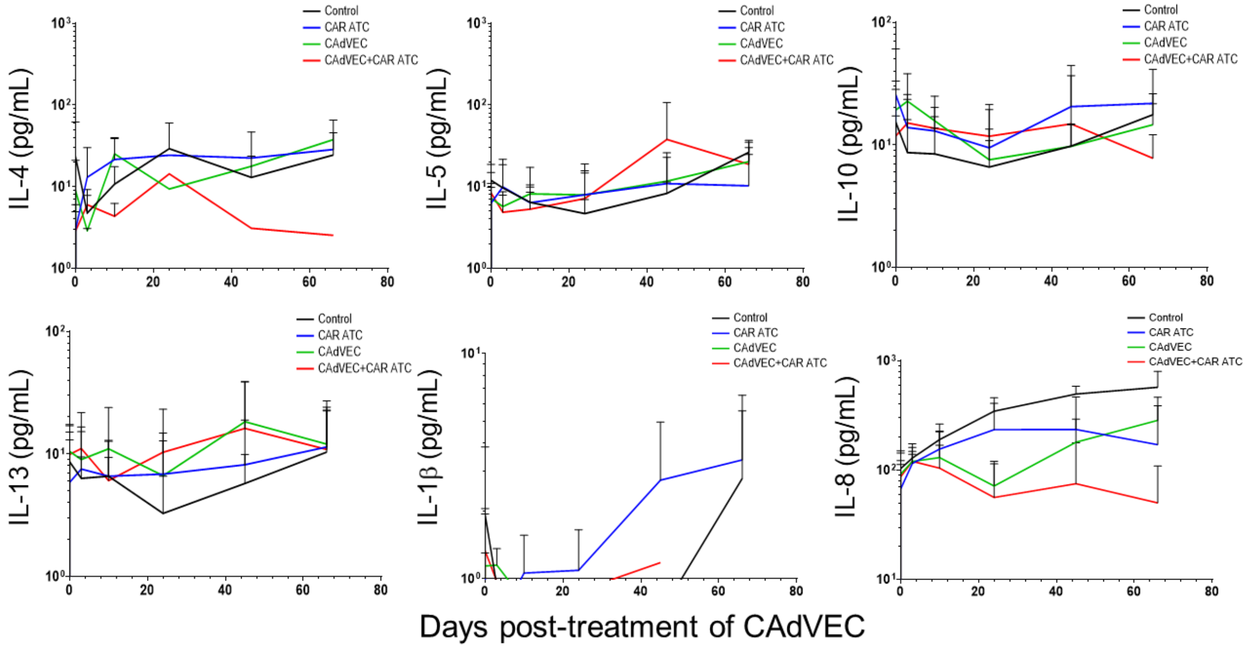

Supplementary Figure 9. ***Th2 cytokine expression was not induced by any treatment condition.*** CFPAC-1 cells were transplanted into the right flank of humanized mice (Control, CART alone, CAdTrio alone: n=5 animals, CAdTrio+CART: n=6 animals). A total of $1x10^7$ vp of *CAdTrio* (Onc:HD=1:1) were injected into the tumor. A total of $1x10^6$ HER2.CARTs expressing *ffLuc* were systemically administered 3 days post-injection of CAd*Trio*. Serum samples were collected from mice at 0, 3, 10, 24, 45, and 66 days post-injection of CAd*Trio*, and human Th1 and Th2 cytokine levels in serum were measured by Multiplex. Data are presented as means ± SD.

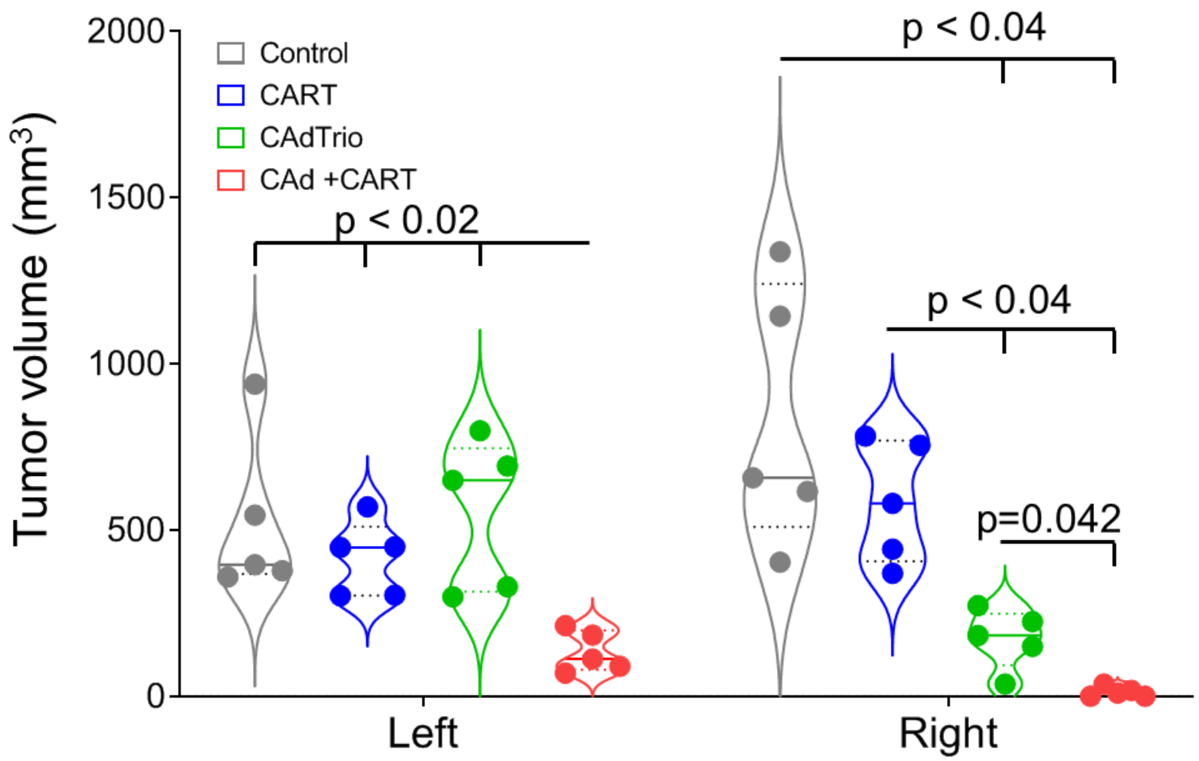

Supplementary Figure 10. ***Combination immunotherapy significantly controls CFPAC-1 tumor growth in humanized mice with multiple tumors.*** CFPAC-1 cells were transplanted into the right and left flanks of humanized mice (n=5 animals). A total of $1x10^7$ vp of CAd*Trio* (Onc:HD=1:1) were injected into the right tumor. A total of $1x10^6$ HER2.CARTs expressing *ffLuc* were systemically administered 3 days post-injection of CAd*Trio*. Tumor volumes shown here from day 31 post injection of CAd, individual data points are represented in violin plot with means ± SD. P-values were determined using ordinary one-way ANOVA with Tukey multiple comparisons, $p < 0.02$ (F(1.977, 7.908)=7.426), $p < 0.04$, p=0.042 (F(1.511, 6.046)=18.35). Statistical significance set at $p < 0.05$, ns > 0.05.

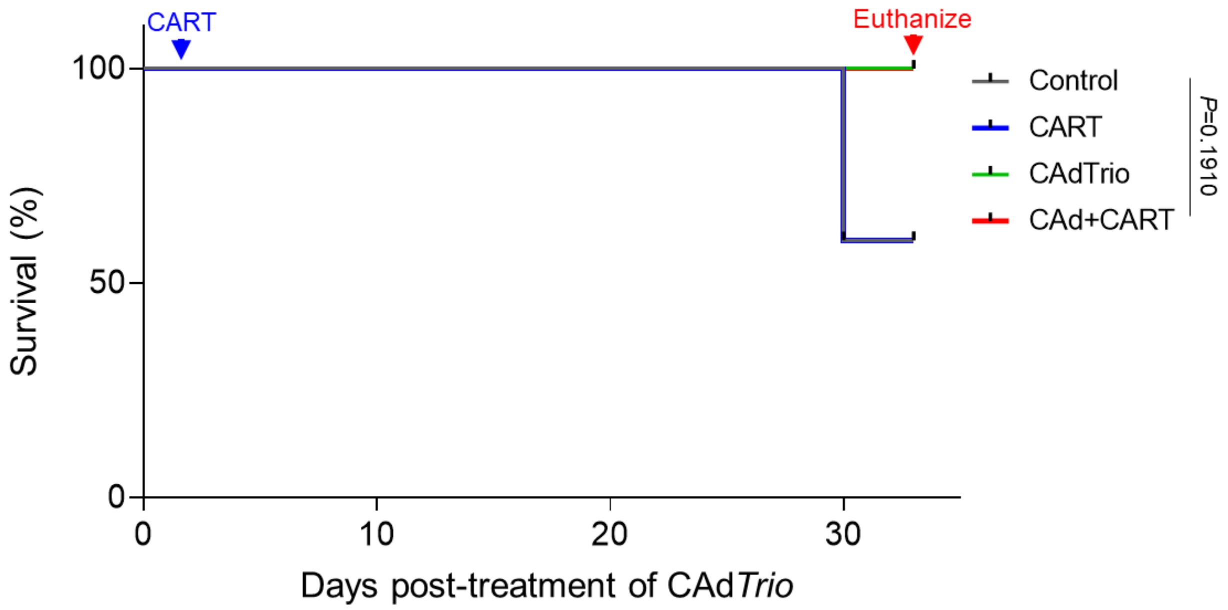

Supplementary Figure 11. ***Kaplan-Meier survival curve in humanized mice with multiple tumors.*** CFPAC-1 cells were transplanted into the right and left flanks of humanized mice (n=5 animals). A total of $1x10^7$ vp of CAd*Trio* (Onc:HD=1:1) were injected into the right tumor. A total of $1x10^6$ HER2.CARTs expressing *ffLuc* were systemically administered 3 days post-injection of CAd*Trio*. Kaplan-Meier survival curve based on the timepoint at which tumor volumes necessitated euthanasia (> 1,500 mm3) after CAd*Trio* administration in mice, p=0.1910. P-values were determined using the Log-rank Mantel-Cox test (dF=3). Statistical significance set at $p < 0.05$, ns > 0.05.

❖ Left tumor

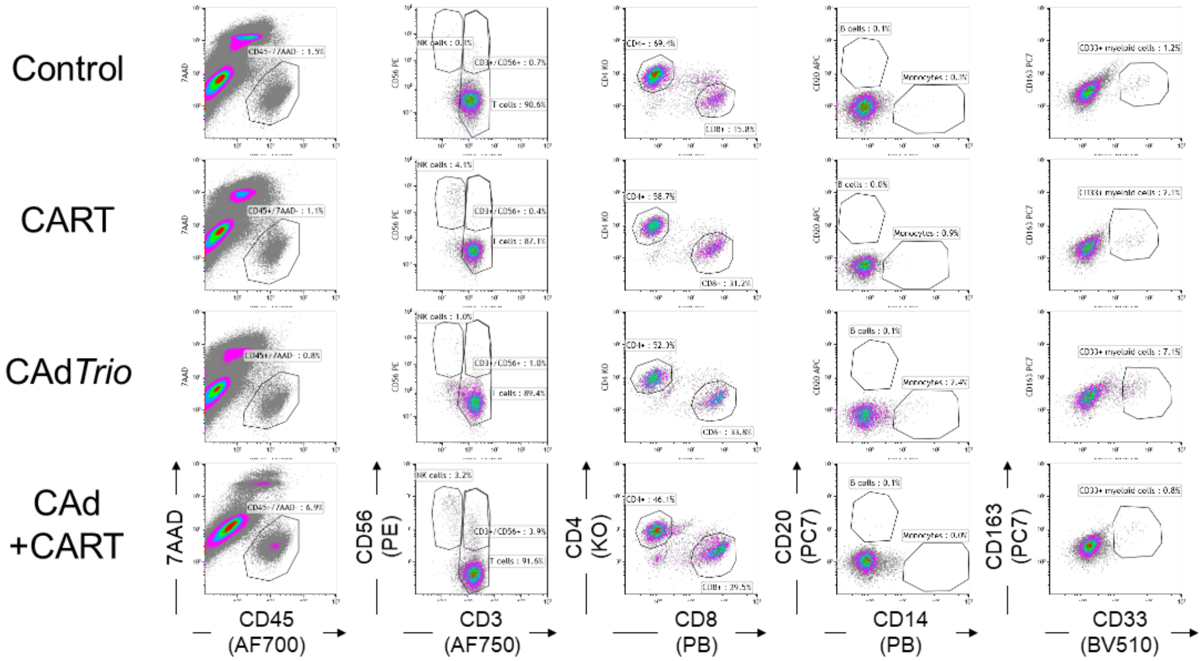

Supplementary Figure 12. ***Combination immunotherapy enhances immune infiltration to untreated tumor site in humanized mice with multiple PDAC tumors*****.** CFPAC-1 cells were transplanted into the right and left flanks of humanized mice (n=5 animals). A total of $1x10^7$ vp of CAd*Trio* (Onc:HD=1:1) were injected into the right tumor. A total of $1x10^6$ HER2.CARTs expressing *ffLuc* were systemically administered 3 days post-injection of CAd*Trio*. CFPAC-1 tumors were harvested from humanized mice at 31 days post-injection of CAd*Trio*, and tumor infiltrating human immune cells were analyzed by flow cytometry. Shown here is representative data from one of five animals per group.

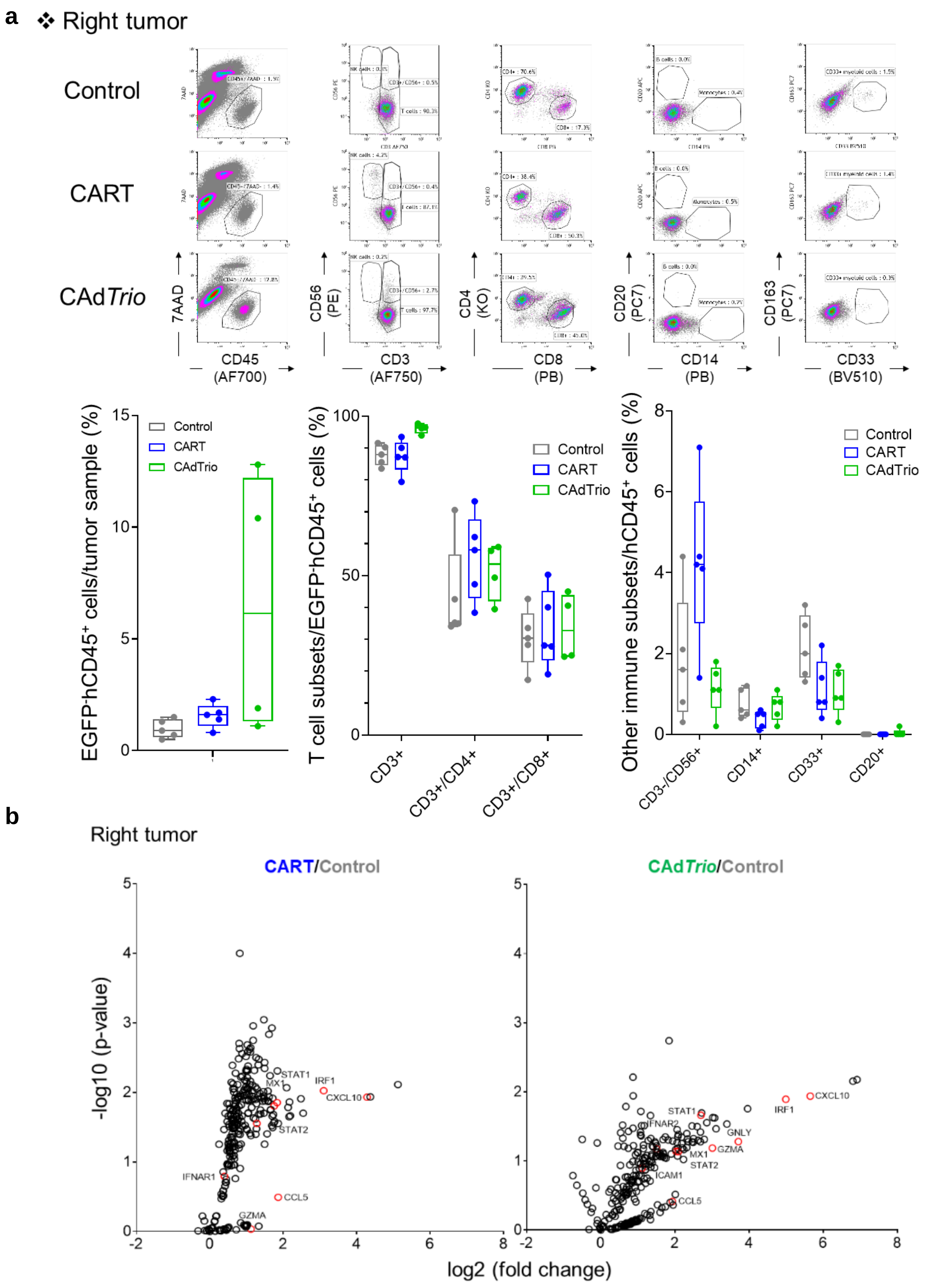
a
Right tumor
Control
CART
CAdTrio
7AAD
CD45 (AF700)
CD56 (PE)
CD3 (AF750)
CD4 (KO)
CD8 (PB)
CD20 (PC7)
CD14 (PB)
CD163 (PC7)
CD33 (BV510)
EGFP-hCD45+ cells/tumor sample (%)
T cell subsets/EGFP-hCD45+ cells (%)
CD3+
CD3+/CD4+
CD3+/CD8+
Other immune subsets/hCD45+ cells (%)
CD3-/CD56+
CD14+
CD33+
CD20+
b
Right tumor
CART/Control
CAdTrio/Control
-log10 (p-value)
log2 (fold change)
STAT1
IRF1
MX1
CXCL10
STAT2
IFNAR1
CCL5
GZMA
IFNAR2
GNLY
ICAM1

Supplementary Figure 13. ***Local combination immunotherapy treatment does not alter infiltration of immune cell subsets*****.** CFPAC-1 cells were transplanted into the right and left flanks of humanized mice (n=5 animals). A total of $1x10^7$ vp of CAd*Trio* (Onc:HD=1:1) were injected into the right tumor. A total of $1x10^6$ HER2.CARTs expressing *ffLuc* were systemically administered 3 days post-injection. **(a)** CFPAC-1 tumors were harvested from humanized mice at 31 days post-injection of CAd*Trio*, and tumor infiltrating human immune cells were analyzed with flow cytometry and **(b)** total RNA was extracted from whole tumor. Gene expression was profiled with Nanostring. Genes showing more than 75% coefficient of variation (CV) compared to control tumors are shown. Box plot elements: central line, median; box limit, upper and lower quartile; whisker, 1.5x inter-quartile range; points, outliers.

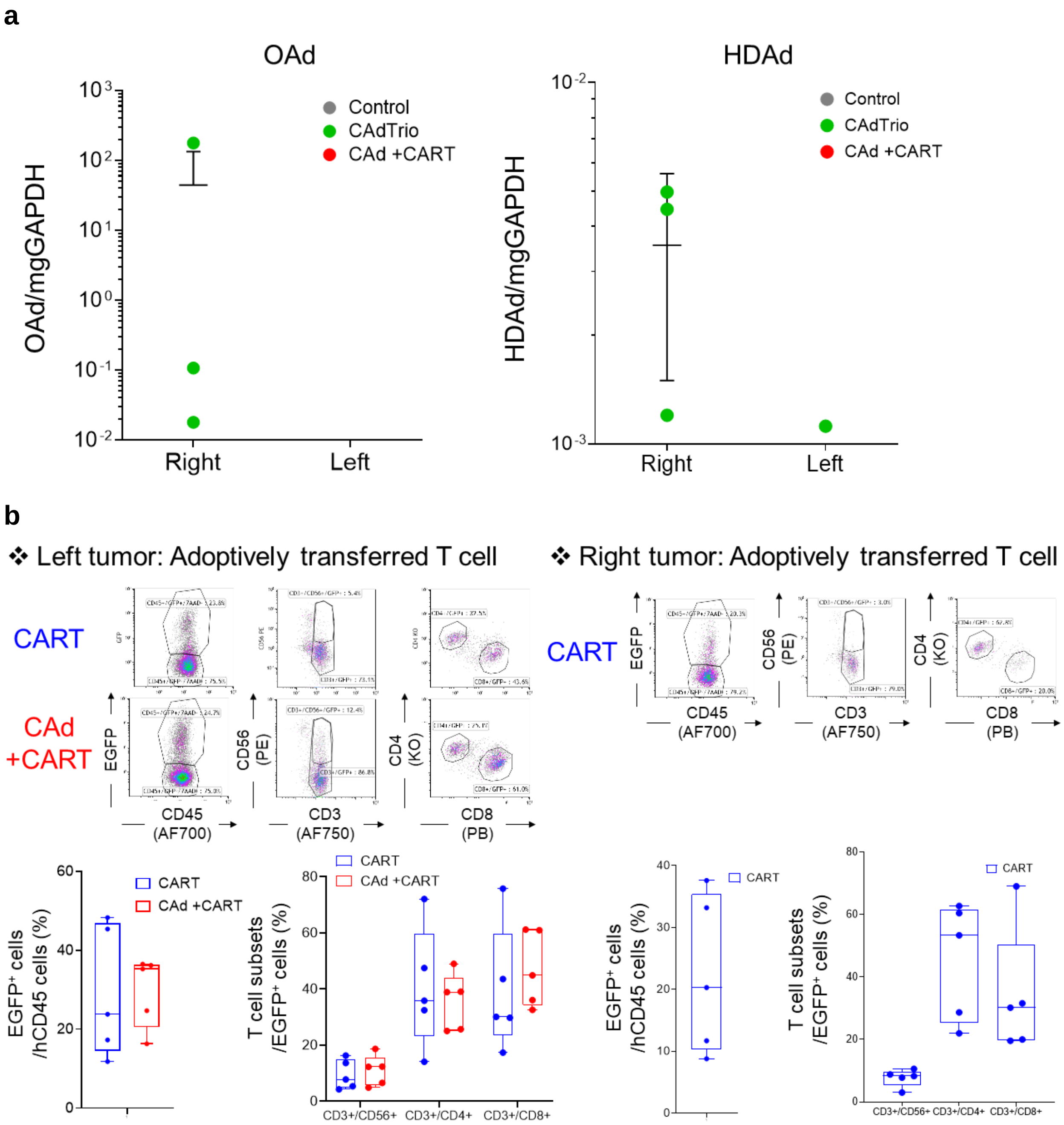

Supplementary Figure 14. ***Residual Ad vectors and HER2.CART at tumor sites*.** CFPAC-1 cells were transplanted into the right and left flanks of humanized mice (n=5 animals). A total of 1x10^7 vp of *CAdTrio* (Onc:HD=1:1) were injected into the right tumor. A total of 1x10^6 HER2.CARTs expressing *ffLuc* were systemically administered 3 days post-injection of CAd*Trio*. Tumors were harvested 31 days post-injection of CAd*Trio*. **(a)** Total DNA was extracted from both right and left tumors separately, and residual Ad vectors were quantified and normalized with murine genomic GAPDH. **(b)** Adoptively transferred HER2.CARTs ($EGFP^+$) at both tumor sites were separately analyzed by flow cytometry. Box plot elements: central line, median; box limit, upper and lower quartile; whisker, 1.5x inter-quartile range; points, outliers.

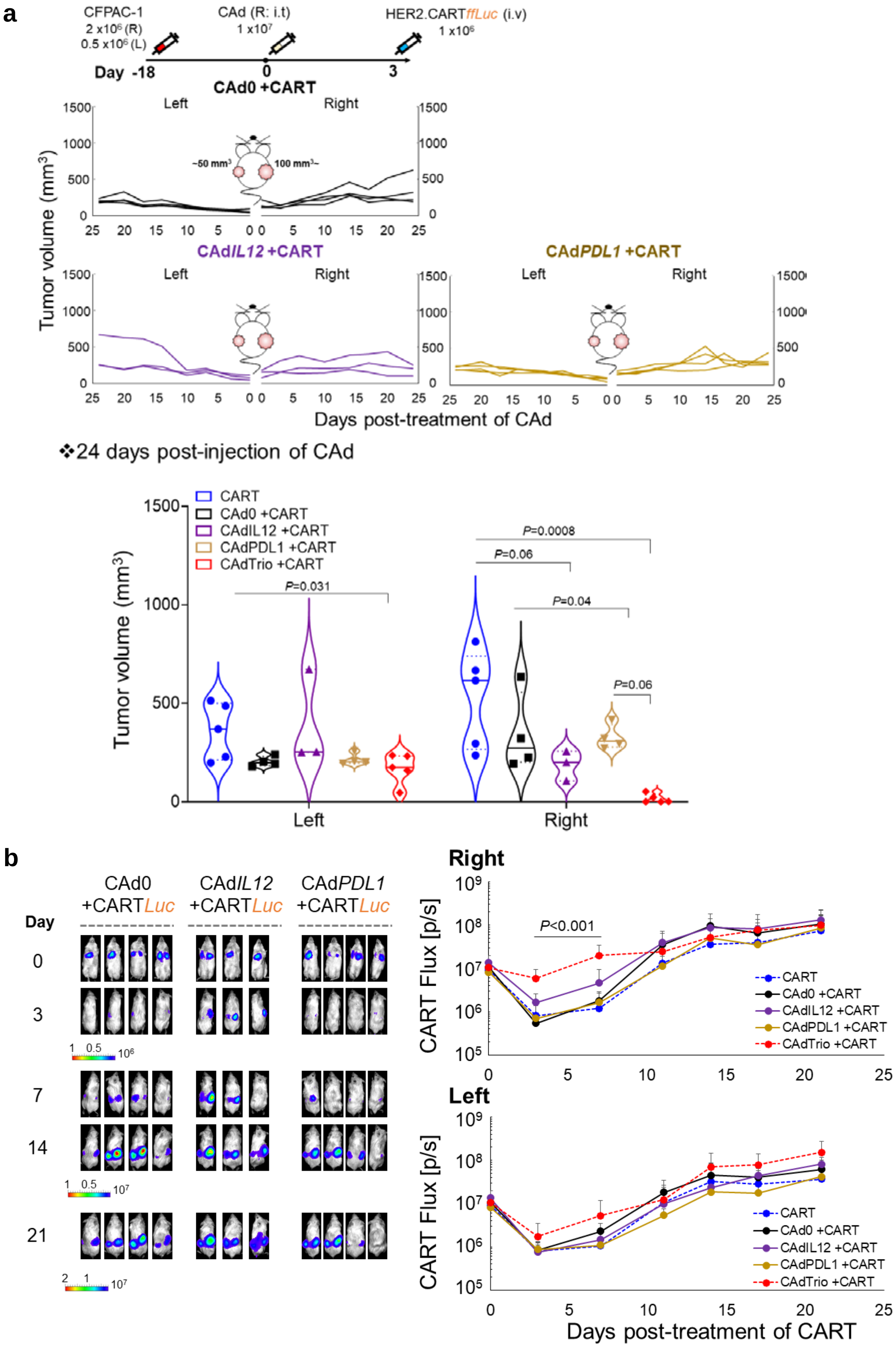

a
CFPAC-1
2 x10^6 (R)
0.5 x10^6 (L)
CAd (R: i.t)
1 x10^7
HER2.CARTffLuc (i.v)
1 x10^6
Day -18
0
3
CAd0 +CART
Left
Right
~50 mm³
100 mm³~
Tumor volume (mm³)
CAdIL12 +CART
CAdPDL1 +CART
Days post-treatment of CAd
❖24 days post-injection of CAd
CART
CAd0 +CART
CAdIL12 +CART
CAdPDL1 +CART
CAdTrio +CART
P=0.031
P=0.0008
P=0.06
P=0.04
P=0.06
Tumor volume (mm³)
Left
Right
b
CAd0 +CARTLuc
CAdIL12 +CARTLuc
CAdPDL1 +CARTLuc
Day
0
3
7
14
21
Right
Left
P<0.001
CART Flux [p/s]
CART
CAd0 +CART
CAdIL12 +CART
CAdPDL1 +CART
CAdTrio +CART
Days post-treatment of CART

Supplementary Figure 15. ***CAd components differently contribute to adoptively transferred HER2.CARTs in humanized mice with multiple PDAC tumors*****. (a)** CFPAC-1 cells were transplanted into the right and left flanks of humanized mice (n=3 (CAdIL12) or 4 (CAd0, CAdPDL1) animals). A total of $1x10^7$ vp of CAd*s* (OAd:HD=1:1) were injected into the right tumor. A total of $1x10^6$ HER2.CARTs expressing *ffLuc* were systemically administered 3 days post-injection of CAd*s*. Tumor volumes were monitored at different time points. Tumor volumes shown here are from day 24 post injection of CAd, individual data points are represented in violin plot with means $\pm$ SD. For comparison, tumor volumes from CART and CAdTrio+CART treated mice at day 24 post injection of CAd from **Figure 5** are shown. P-value was determined using unpaired two-tailed T test for left tumors. $p < 0.031$ (t=2.597, dF=8). P-values were determined using ordinary one-way ANOVA with Tukey multiple comparisons for right tumors, p=0.0008 (DF=16), p=0.06 (DF=16), p=0.04 (DF=16), p=0.06 (DF=16). Statistical significance set at $p < 0.05$, ns > 0.05. **(b)** Bioluminescence of HER2.CARTs was monitored at different time points. For comparison, bioluminescence data of CART and CAdTrio+CART treated mice from **Figure 5** are shown here, denoted by dashed lines. Data are presented as means $\pm$ SD. P-values were determined using ordinary one-way ANOVA with Tukey multiple comparisons, p<0.001 (F=8.641). Statistical significance set at $p < 0.05$, ns > 0.05.

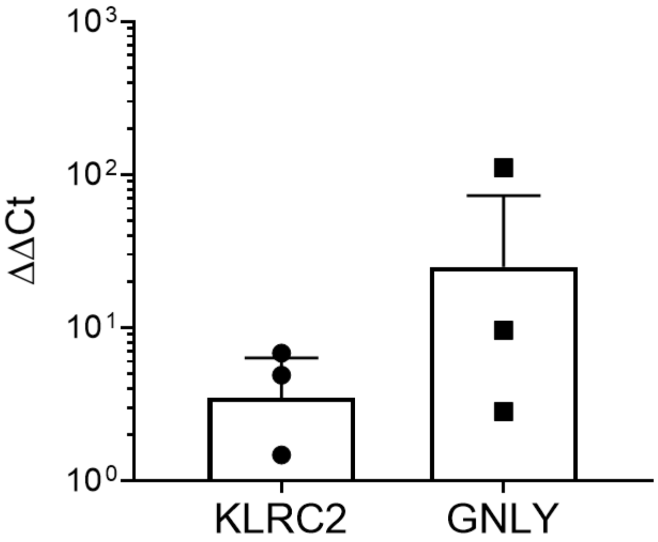

Supplementary Figure 16. ***CAdTrio induces KLRC2 and GNLY expression at early time points*. (a)** CFPAC-1 cells were transplanted into the right flank of humanized mice, a total of $1x10^7$ vp of *CAdTrio* (Onc:HD=1:1) were injected into the tumor. CFPAC-1 tumors were harvested from humanized mice (non-humanized mice: n=4 animals, humanized mice: n=5 animals) at 3 days post-injection of CAd*Trio*, and total RNA was extracted from whole tumors. KLRC2 and GNLY genes were quantified and normalized with human β-Actin. Data are presented as means ± SD.

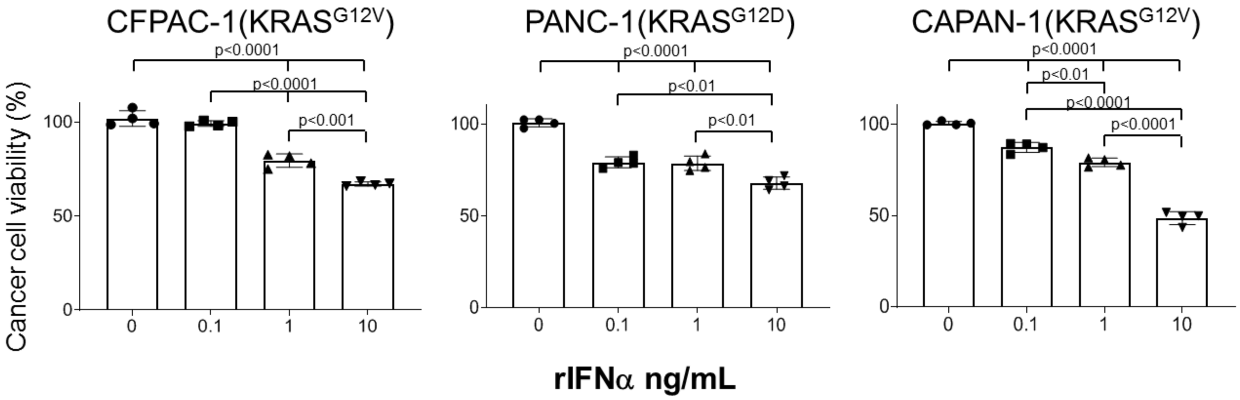

Supplementary Figure 17. ***IFNα induces some PDAC cell death in vitro***. PANC-1, CAPAN-1 and CFPAC-1 expressing ffLuc were cultured with increasing doses of rIFNα (n=4 independent biological samples). Cells were harvested 72 hours post-coculture, and viable cells were analyzed by luciferase assay. Data are presented as means ± SD. P-values were determined by ordinary one-way ANOVA with Tukey multiple comparisons. CFPAC ($F(3,12)=132.20$), Panc1 ($F(3,12)=74.83$), CAPAN1 ($F(3,12)=292.9$). Statistical significance set at $p < 0.05$, ns > 0.05.
