# Supplementary material for: Oncolytic adeno-immunotherapy modulates the immune system enabling CAR T-cells to cure pancreatic tumors
Source: Commun Biol. 2021 Mar 19;4:368. doi: 10.1038/s42003-021-01914-8 (PMC7979740; doi:10.1038/s42003-021-01914-8)
Supplement: Supplementary file 3 — Description of Additional Supplementary Files [file 42003_2021_1914_MOESM3_ESM.pdf]

Description of Additional Supplementary Files

File name: Supplemental Data 1
Description: Nanostring Advanced Analysis from Left Tumor.

File name: Supplemental Data 2
Description: Nanostring Advanced Analysis from Right Tumor.

File name: Supplemental Data 3
Description: Nanostring Advanced Analysis comparing combination treatment to single agent treatments.

File name: Supplemental Data 4
Description: All source data underlying the graphs and charts presented in the main figures.
