# Supplementary material for: Oncolytic adeno-immunotherapy modulates the immune system enabling CAR T-cells to cure pancreatic tumors
Source: Commun Biol. 2021 Mar 19;4:368. doi: 10.1038/s42003-021-01914-8 (PMC7979740; doi:10.1038/s42003-021-01914-8)
Supplement: Supplementary file 8 — Reporting Summary [file 42003_2021_1914_MOESM8_ESM.pdf]

nature research

Corresponding author(s): Masataka Suzuki

Last updated by author(s): Sep 23, 2020

Reporting Summary

Nature Research wishes to improve the reproducibility of the work that we publish. This form provides structure for consistency and transparency in reporting. For further information on Nature Research policies, see our Editorial Policies and the Editorial Policy Checklist.

Statistics

For all statistical analyses, confirm that the following items are present in the figure legend, table legend, main text, or Methods section.

n/a	Confirmed	
☐	☒	The exact sample size (*n*) for each experimental group/condition, given as a discrete number and unit of measurement
☐	☒	A statement on whether measurements were taken from distinct samples or whether the same sample was measured repeatedly
☐	☐	The statistical test(s) used AND whether they are one- or two-sided<br>*Only common tests should be described solely by name; describe more complex techniques in the Methods section.*
☐	☒	A description of all covariates tested
☐	☒	A description of any assumptions or corrections, such as tests of normality and adjustment for multiple comparisons
☐	☒	A full description of the statistical parameters including central tendency (e.g. means) or other basic estimates (e.g. regression coefficient) AND variation (e.g. standard deviation) or associated estimates of uncertainty (e.g. confidence intervals)
☐	☒	For null hypothesis testing, the test statistic (e.g. *F*, *t*, *r*) with confidence intervals, effect sizes, degrees of freedom and *P* value noted<br>*Give P values as exact values whenever suitable.*
☒	☐	For Bayesian analysis, information on the choice of priors and Markov chain Monte Carlo settings
☒	☐	For hierarchical and complex designs, identification of the appropriate level for tests and full reporting of outcomes
☒	☐	Estimates of effect sizes (e.g. Cohen's *d*, Pearson's *r*), indicating how they were calculated

Our web collection on statistics for biologists contains articles on many of the points above.

Software and code

Policy information about availability of computer code

Data collection	Image Studio v5.2, Gallios software 1.1, Living Image v4.5.2, Omega v5.11, Magellan v7.0, nSolver4.0
Data analysis	Kaluza v1.2 , nSolver4.0, CFX96 manager software v3.1, Graphpad Prism v8.0, Living Image v4.5.2, Microsoft excel 2016

For manuscripts utilizing custom algorithms or software that are central to the research but not yet described in published literature, software must be made available to editors and reviewers. We strongly encourage code deposition in a community repository (e.g. GitHub). See the Nature Research guidelines for submitting code & software for further information.

Data

Policy information about availability of data

All manuscripts must include a data availability statement. This statement should provide the following information, where applicable:

- Accession codes, unique identifiers, or web links for publicly available datasets
- A list of figures that have associated raw data
- A description of any restrictions on data availability

The authors declare that the data supporting the findings of this study are available within the paper and its supplementary information files.

Field-specific reporting

Life sciences study design

All studies must disclose on these points even when the disclosure is negative.

Sample size	All group sizes described are based on differences among treatment groups assumed to reach conventional levels of significance (p<0.05) on analysis based on estimates from pilot experiments and previously published results.
Data exclusions	No data were excluded from analyses
Replication	In vitro experiments were conducted with at least triplicate technical replicates and repeated to confirm independent results and reproducibility. In vivo experiments were typically conducted twice with the exception of the experiment corresponding to supplementary fig 14 due to lack of availability of humanized mice.
Randomization	Animals were randomly assigned to treatment groups for all experiments.
Blinding	Where possible analysis was blinded, however, due to restrictions in our biohazard protocol some analysis for in vivo experiments were not blinded (e.g. tumor volume) because cages must be labeled with treatment and due to limited access of personnel handling biohazard treated animals. Samples submitted to core facilities (for IHC and Nanostring) were blinded.

Reporting for specific materials, systems and methods

We require information from authors about some types of materials, experimental systems and methods used in many studies. Here, indicate whether each material, system or method listed is relevant to your study. If you are not sure if a list item applies to your research, read the appropriate section before selecting a response.

Materials & experimental systems

n/a	Involved in the study	
☐	☒	Antibodies
☐	☒	Eukaryotic cell lines
☒	☐	Palaeontology and archaeology
☐	☒	Animals and other organisms
☒	☐	Human research participants
☐	☐	Clinical data
☒	☐	Dual use research of concern

Methods

n/a	Involved in the study	
☒	☐	ChIP-seq
☐	☒	Flow cytometry
☒	☐	MRI-based neuroimaging

Antibodies

Antibodies used	HA epitope tag antibody (26183), For Human Flow cytometry: 7AAD (BD Biosciences #559925), IgG-Fc-AF647 (Southern Biotech #2014-31, polyclonal), CD11b-APC (Biolegend #301310, clone ICRF44), CD14-PB (Biolegend #367121, clone 63D3), CD163-PE/Cy7 (Biolegend #326513, clone RM3/1), CD20-PE/Cy7 (Beckman Coulter #IM3629, clone B9E9), CD20-APC (BD Biosciences #340941, clone L27), CD3-AF750 (Beckman Coulter #A66329, clone UCHT1), CD33-BV510 (Biolegend #366609, clone P67.6), CD4-KO (Beckman Coulter #A96417), CD45-AF700 (Beckman Coulter #A71117, clone J33), CD56-PE (Beckman Coulter #IM2073U, clone N901), CD56-FITC (BD Biosciences #340410, clone NCAM16.2), CD8-APC (Beckman Coulter #IM2469U, clone B9.11), CD8-PB (Beckman Coulter #A82791, clone B9.11), HER2-APC (Biolegend #324408, clone 24D2), HLA-A2-FITC (BD Biosciences #551285, clone BB7.2), HLA-DR-PB (Beckman Coulter #A74781, clone Immu-357), Human TruStain FcX (Biolegend #422302), NKG2C-PE (R&D Systems #FAB138P-025, clone 134591), Recombinant HER2 Fc Chimera (R&D Systems #1129-ER-050)
Validation	26183 is specific for the HA peptide YPYDVPDYA. The antibody has been used successfully in Western blot and immunoprecipitation applications. All antibodies used for flow cytometry were validated by the manufacturer directly.

Eukaryotic cell lines

Policy information about cell lines

Cell line source(s)	We obtained human pancreatic lines CAPAN-1, CFPAC-1 and PANC-1 from ATCC (Manassas, VA) in 2018
Authentication	Cell lines were authenticated with Short Tandem Repeat (STR) profiling by ATCC
Mycoplasma contamination	All cell lines used tested negative for mycoplasma.
Commonly misidentified lines (See ICLAC register)	*Name any commonly misidentified cell lines used in the study and provide a rationale for their use.*

Animals and other organisms

Policy information about studies involving animals; ARRIVE guidelines recommended for reporting animal research

Laboratory animals	All animal experiments were carried out in mice: 7-8 week old, male and female NOD.Cg-Prkdcscid Il2rgtm1Wjl/SzJ (NSG) were used for xenograft tumor engraftment. 1-2 day old, male and female, NOD.Cg-Prkdcscid Il2rgtm1Wjl Tg(CMV-IL3,CSF2,KITLG)1Eav/MloySzJ (NSGSGM3) were sublethally irradiated (100 cGy) and intrahepatically injected with 5 x104 human cord-blood unit (CBU)-derived CD34+ cells, 8-9 weeks post-injection mice were used for tumor engraftment
Wild animals	*Provide details on animals observed in or captured in the field; report species, sex and age where possible. Describe how animals were caught and transported and what happened to captive animals after the study (if killed, explain why and describe method; if released, say where and when) OR state that the study did not involve wild animals.*
Field-collected samples	*For laboratory work with field-collected samples, describe all relevant parameters such as housing, maintenance, temperature, photoperiod and end-of-experiment protocol OR state that the study did not involve samples collected from the field.*
Ethics oversight	The Baylor College of Medicine Institutional Animal Care and Use Committee approved all animal experiments.

Note that full information on the approval of the study protocol must also be provided in the manuscript.

Clinical data

Policy information about clinical studies

All manuscripts should comply with the ICMJEguidelines for publication of clinical research and a completedCONSORT checklist must be included with all submissions.

Clinical trial registration	*Provide the trial registration number from ClinicalTrials.gov or an equivalent agency.*
Study protocol	*Note where the full trial protocol can be accessed OR if not available, explain why.*
Data collection	*Describe the settings and locales of data collection, noting the time periods of recruitment and data collection.*
Outcomes	*Describe how you pre-defined primary and secondary outcome measures and how you assessed these measures.*

Flow Cytometry

Plots

Confirm that:

- [x] The axis labels state the marker and fluorochrome used (e.g. CD4-FITC).
- [x] The axis scales are clearly visible. Include numbers along axes only for bottom left plot of group (a 'group' is an analysis of identical markers).
- [x] All plots are contour plots with outliers or pseudocolor plots.
- [x] A numerical value for number of cells or percentage (with statistics) is provided.

Methodology

Sample preparation	For in vitro samples, cells were harvested and washed with facs buffer prior to staining. For in vivo samples, tumors were rinsed with PBS, minced, and incubated in RPMI media containing human tumor dissociation reagents (Miltenyi Biotech Inc.) at 37ºC for 1 hour. Cells were passed through a 70-μm cell strainer (BD Pharmingen), and murine stroma cells were removed using a Mouse Cell Depletion kit (Miltenyi Biotech Inc.).
Instrument	Gallios (Beckman)
Software	Gallios software was used to collect data which was analyzed with Kaluza software.
Cell population abundance	In sorting GFP positive cells, only the brightest 20% of cells were sorted, uniformity of GFP expression was subsequently confirmed by flow cytometry.
Gating strategy	After FSC/SSC and live cell gating all gates were determined based on isotype control staining.

- [ ] Tick this box to confirm that a figure exemplifying the gating strategy is provided in the Supplementary Information.
